# A Core Set of Snap Bean Genotypes Established by Phenotyping a Large Panel Collected in Europe

**DOI:** 10.3390/plants11050577

**Published:** 2022-02-22

**Authors:** Carmen García-Fernández, Maria Jurado, Ana Campa, Creola Brezeanu, Valérie Geffroy, Elena Bitocchi, Roberto Papa, Juan Jose Ferreira

**Affiliations:** 1 Plant Genetic Group, Regional Service for Agrofood Research and Development (SERIDA), 33300 Villaviciosa, Spain; cgarcia@serida.org (C.G.-F.); mjurado@serida.org (M.J.); acampa@serida.org (A.C.); 2 Stațiunea de Cercetare Dezvoltare Pentru Legumicultură, 600388 Bacău, Romania; creola.brezeanu@legumebac.ro; 3 Université Paris-Saclay, CNRS, INRAE, Univ Evry, Institute of Plant Sciences Paris-Saclay (IPS2), 91405 Orsay, France; valerie.geffroy@universite-paris-saclay.fr; 4 Department of Agricultural, Food and Environmental Sciences, Marche Polytechnic University, Via Brecce Bianche, 60131 Ancona, Italy; e.bitocchi@staff.univpm.it (E.B.); r.papa@univpm.it (R.P.)

**Keywords:** *Phaseolus vulgaris* L., diversity, phenotyping, genotyping, QTL validation, core collections, pod traits, diversity

## Abstract

Snap beans are a group of bean cultivars grown for their edible immature pods. The objective of this work was to characterize the diversity of pod phenotypes in a snap bean panel (SBP), comprising 311 lines collected in Europe, and establish a core set (Core-SBP) with the maximum diversity of pod phenotypes. Phenotyping of the SBP was carried out over two seasons based on 14 quantitative pod dimension traits along with three qualitative traits: pod color, seed coat color, and growth habit. Phenotypes were grouped into 54 classes using a hierarchical method, and a Core-SBP with one line per phenotype class was established. A further field-based evaluation of the Core-SBP revealed higher diversity index values than those obtained for the SBP. The Core-SBP was also genotyped using 24 breeder-friendly DNA markers tagging 21 genomic regions previously associated with pod trait control. Significant marker-trait associations were found for 11 of the 21 analyzed regions as well as the locus fin. The established Core-SBP was a first attempt to classify snap bean cultivars based on pod morphology and constituted a valuable source of characteristics for future breeding programs and genetic analysis.

## 1. Introduction

Snap beans, (syn. garden, French, or green beans) are a group of common bean cultivars (*Phaseolus vulgaris* L.), whose fresh pods (immature pods and seeds) are consumed as green vegetables. Fresh pods are harvested at a physiologically immature stage of development, when the full length has been reached but the pod filling process is at an early-intermediate stage (beginning of stage R8 [1]). One trait that is highly homogeneous among snap beans is the low content of lignin in the pods, which makes most modern snap bean cultivars fully indehiscent [2]. Snap bean pods are comprised of 90% water and a small percentage of carbohydrates and proteins. However, they are nutritionally interesting for their content of dietary fiber, vitamins (folates, A, B, and C), and essential minerals such as K, Ca, Fe, Mg, Mn, P, and Zn. In addition, the bean pods contain phenols and flavonoids, two families of molecules well known for their antioxidant action [3,4]. These molecules play an important role in human health, because they possess antioxidant activity, which has anti-diabetic, anti-obesity, anti-inflammatory, anti-mutagenic, and anti-carcinogenic properties [5].

The domestication of common beans took place in central and south America, where the wild forms can still be found. Two major eco-geographically and genetically distinct gene pools, the Andean and the Mesoamerican, have been described for common beans [6,7] (see [8] for a review). American landraces with edible fresh pods are rarely found [9]. Snap bean cultivars may have arisen as a result of selective pressures on pod characteristics exerted on dry cultivars, which are mainly consumed as mature seeds after rehydration and cooking [10]. The dry bean cultivars can be consumed during the early-developmental stages when the pod fiber content is low, particularly during periods when food is scarce [11]. Although the origin of the snap bean is uncertain, snap bean cultivars and breeding strategies were reported in the early 20th century in America. For example, Wade [12] reported that the cultivation of older varieties from the US, including cv ‘Tendergreen’, ‘Stringless Green Refugee’, and ‘Red Velentine’, dates back to the early 20th century. In Europe, small-scale seed companies and cultivation in small orchards have contributed to maintaining snap bean diversity. Snap bean varieties such as ‘Fin de Bagnols’, ‘Triomphe de Farcy’, ‘Merveille du Marché’, ‘Merveille de Venise’, and ‘Roi des Beurres’ were reported during the first half of the 20th century [13]. Furthermore, Puerta Romero [14] described 120 snap beans in a set of 296 accessions collected in the middle of the 20th century in Spain (e.g., cv ‘Garrafal oro’), and indicated that 49 of these 120 snap beans were used both as fresh pods and dry seeds.

Common beans exhibit high levels of morphological diversity in their pods (see [15]) and seeds (e.g., [16,17]). Phenotypic diversity is observed in pod size, shape (in length and cross-section), tip shape, number of seeds in each pod (NSP), color and color distribution, presence of fibrous inedible strings along the seams, and potential use for human consumption (shell/edible). Based on the pod phenotype, different market classes have been established: (i) ‘String snap bean’, referring to pods from which the suture strings must be removed before consumption; (ii) ‘Yellow wax’ and ‘Green bean’, referring to yellow and green pods, respectively; (iii) ‘Romano type’, popularly known as ‘Italian green beans’, with very large and flat pods; (iv) ‘Blue Lake type’ with dark green pods that remain stringless and fiberless [11]; (v) ‘Filet type’, also called ‘French green beans’ or ‘Haricot verts’, with long, round, straight and very slim pods [18,19]; and (vi) ‘Garrafal type’, with green hook-shaped and very large pods with a pear-shaped cross-section [14]. However, these classifications do not allow for all of the diversity in pods to be detailed. There are many more phenotypic variations of snap beans, such as various pod color patterns and short and very flat pods, among others.

Molecular marker analysis has revealed that snap bean cultivars can be assigned to both the Andean and Mesoamerican gene pools, and many cultivars exhibit different levels of admixture between these gene pools [10,17,19,20]. For instance, in a study by Wallace et al. [11], snap bean genotypes of the market class ‘Blue lake’ were shown to belong to both the Andean and Mesoamerican gene pools, and several of them exhibited admixture between the gene pools. The Spanish Diversity Panel includes 60 well-known snap beans, and 34 of them are grouped in a cluster close to the Andean gene pool, with different levels of Mesoamerican introgression [17]. Snap beans are an interesting group that includes landraces and elite cultivars that have adapted to multiple environments and requirements due to selection and breeding efforts that have included recombination between these gene pools.

Most studies of diversity in common beans have been focused on dry beans, because they represent most of the varieties used for cropping in this species and offer a very important source of vegetable protein worldwide (http://www.fao.org/faostat/, accessed on 15 November 2021). The few studies to have investigated the genetic diversity of snap beans have been based on variation provided by molecular markers (e.g., [11,19,20]). Pod phenotype is a relevant trait in snap beans, because it influences consumer choice and their potential use as fresh or processed produce (i.e., frozen, canned [18,21]). Tools for high-throughput phenotyping are now available to record phenotypic variation in detail [22]. The main objective of this work was to describe the phenotypic diversity in pods from a panel of snap beans (SBP) composed of 311 accessions collected from European gene banks, working collections, and seed companies to establish a core set (Core-SBP) with maximum phenotypic diversity and minimum redundancy. The usefulness of this core set was investigated by validation with breeder-friendly DNA markers associated with major genomic regions previously identified as controlling the morphological traits of pods.

## 2. Results

### 2.1. Phenotypic Variation

The results of evaluating the phenotypic diversity in 311 snap bean accessions showed wide ranges of variation for the 14 quantitative pod traits evaluated in 311 lines (Table 1 and Table 2; Figure S1). For instance, PL and NSP ranged from 7.3 cm (observed in SBP382) to 25.86 cm (SBP299), and from 3.7 NSP (SBP280) to 8.75 NSP (SBP012), respectively. Similarly, 25-seed weight shows a wide variation in this panel, ranging from 1.92 g (SBP141) to 22.6 g (SBP343). All the evaluated traits exhibited a continuous distribution (Figure S1), although only PSW and NSP showed a good fit to a normal distribution (Shapiro-Wilk normality test). The SBP also showed wide variation for color with green (212 lines), yellow (80), purple (7), green mottled (11), and yellow mottled (1) pods. Concerning seed coat color, most lines had a seed coat color (170 overall), including cream (22), canella (15), brown and dark brown (66), red (4), purple (10), and black (53). There were 141 lines with a white seed main color. Regarding growth habit, 237 lines showed determinate bush habits and 74 indeterminate climbing habits.

Significant correlations were detected between most of the traits evaluated (see Figure S2). Interestingly, significant correlations were found between 25-seed weight and all the variables of pod dimension (except PSW). There was a significant correlation between PSH and PLW, two variables associated with the width of the pod. Correlations were not significant, however, between the values of NSP and PLA, PSP, PSA and PSW, or PSW and the six longitudinal pod traits (PLP, PLA, PLW, PL, PLC, PL/PLC) and PSH (see Table 1).

### 2.2. Hierarchical Clustering on Principal Components

**Figure 1:**
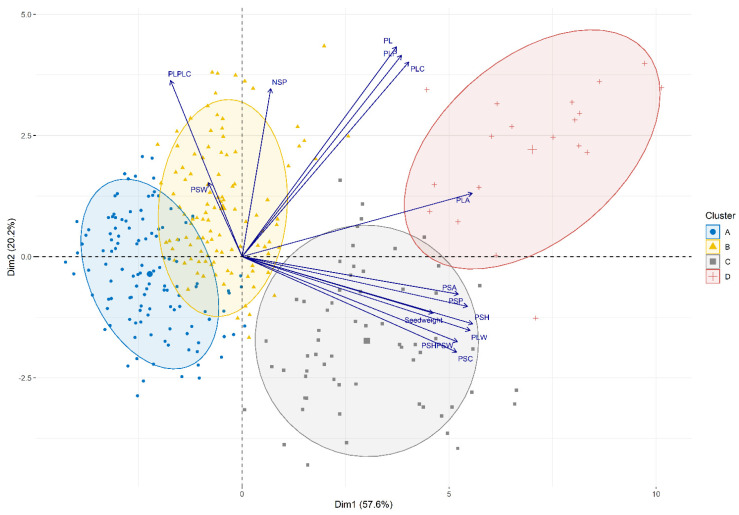
Biplots showing the results of the Hierarchical Clustering on Principal Components analysis from 14 evaluated quantitative pod traits (PLP, PLA, PLW, PL, PLC, PL/PLC, PSP, PSA, PSW, PSH, PSH/PSW, PSC, NSP, Seed weigh; see Table S2) in the Snap Bean Panel. Ellipses representing the clusters were drawn considering a confidence interval > 0.8.

Hierarchical Clustering on Principal Components (HCPC) analysis using the averages of the 14 quantitative morphological pod traits revealed two main dimensions that explained 78% of the variance and established four main clusters (Figure 1):

1. Cluster A included 121 lines with significantly lower values for PL, PLA, PLP, PLW, PSA, PSH, PSP, and 25 seed weight (Table S1). The group included lines with small pods characterized by a round cross-section. Moreover, this group included old and well-known cultivars such as ‘Harvester’, ‘Widusa’, ‘Midas’, ‘Slendergreen’, ‘Beurre de Rocquencourt’, ‘Manteca de los Mercados‘, and ‘Cherokee Trail of Tears’. Most lines in this cluster had determinate growth habits (118).
2. Cluster B included 109 lines with intermediate values for PL, PLA, PLP, PLW, PSA, PSP, PSH, PSW, and 25-seed weight, which were significantly different from those for the other three groups (Table S1). This group exhibited higher values for PSW, indicating lines with pods characterized by a round cross-section or a cross-section like an eight. Well-known cultivars such as ‘Slenderwax’, ‘Improvement Tendergreen’, ‘Topcrop’, ‘Fin de Bagnols’, ‘Gloire de Saumur’, ‘Contender’, ‘La Victorie’, and ‘Tendergreen’ belonged to this cluster.
3. Cluster C included 63 lines with intermediate values for PL, PLA, PLP, PLPLC, PLW, PSA, PSC, PSH, PSHPSW, and PSP that were significantly different from those for the other three groups (Table S1). This group exhibited lower values for the PL/PLC ratio, indicating very straight pods. Interestingly, many lines provided by the KIS (Agricultural Institute of Slovenia, Ljubljana, Slovenia) were grouped into this cluster.
4. Cluster D included 18 lines with significantly higher values for most traits (PL, PLA, PLC, PLP, PLW, PSA, PSC, PSH, PSHPSW, PSP, and NSP). The group included lines with large pods and flat pod cross-sections (Table S1). Within this cluster were lines such as the well-known ‘Garrafal Oro’, a Spanish traditional cultivar, and ‘Musica’ and ‘Marconi’, two Romano types. This cluster only included lines with indeterminate climbing habits.

### 2.3. Establishment of a Core-SBP

**Figure 2:**
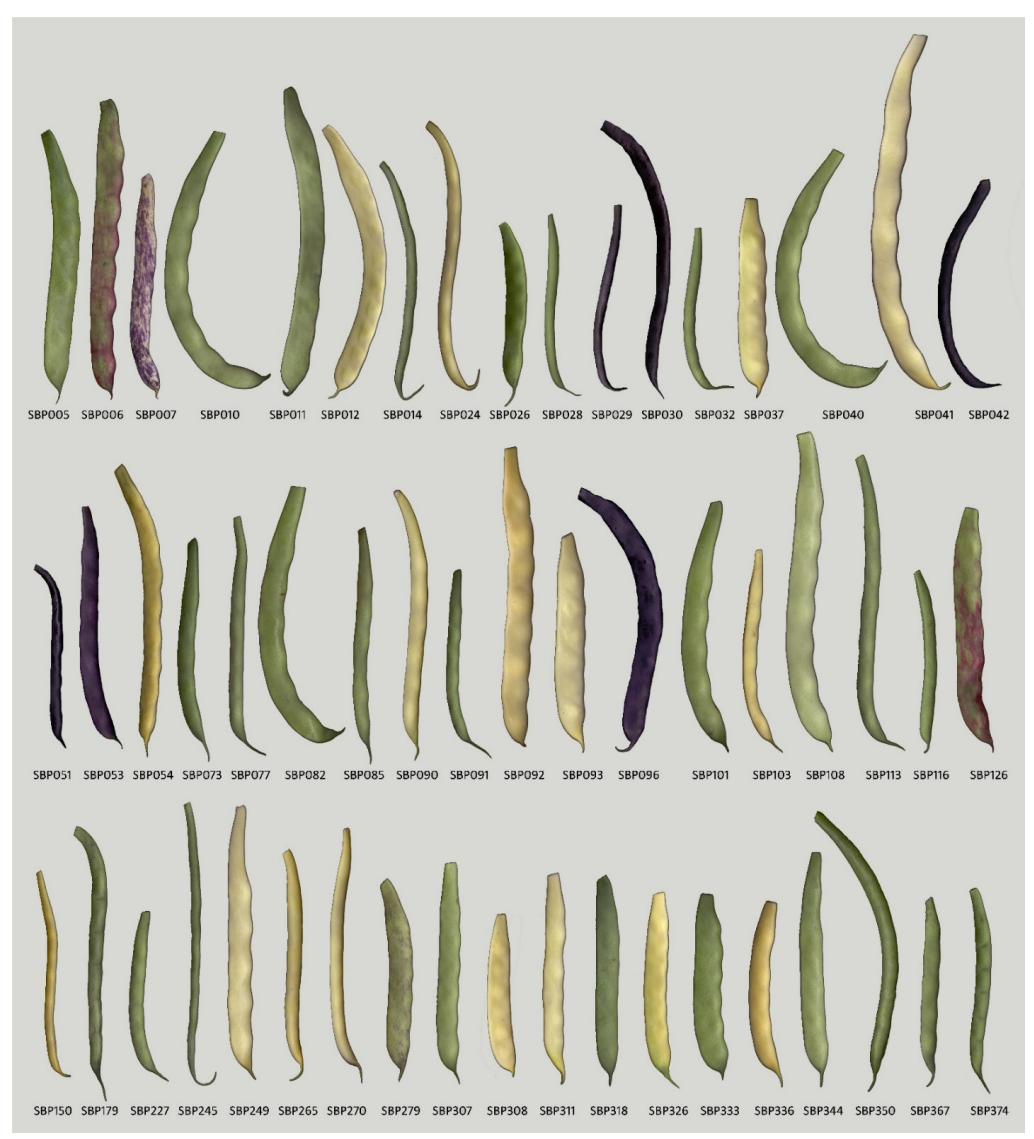
Immature pod phenotype of the 54 lines included in the Core-SBP. Pods were scanned at 300 dpi.

A total of 54 phenotypic classes were established after considering the four clusters obtained by HCPC analysis, five pod colors, and eight main seed coat colors (see Table S2). The largest clustering was that containing beans with short green pods and white seed coats (73 lines included in cluster A), followed by the class of beans with green pods and white seed coats (23 lines included in cluster B), and the class of beans with green pods and black seed coats (20 lines included in cluster B). In contrast, 16 classes were represented by a single line. Cluster C was the most diverse, with 17 classes, followed by cluster A, with 15 classes. From this grouping, one line per phenotypic class was randomly selected to establish the Core-SBP containing 54 lines representing the phenotypic diversity of the SBP (see Table S2; Figure 2). This Core-SBP included 34 lines with determinate growth habits and 20 lines with indeterminate climbing habits.

### 2.4. Core-SBP Evaluation

The Core-SBP was further evaluated in the field during summer 2021 to verify its phenotypic diversity. Data for lines SBP080, SBP265, and SBP326 were not available due to pest damage. The Core-SBP maintained a wide variation for the 12 pod traits, NSP, and 25-seed weights (Table 2). For example, the PL ranged from 7.07 cm (SBP029) to 19.01 cm (SBP041), PSH from 0.66 cm (SPB_150) to 1.91 cm (SBP333), and NSP from 4.1 (SBP318) to 8 (SBP014). HCPC analysis using the phenotypic data collected in the 2021 field study also grouped the Core-SBP lines into four main clusters (see Figure S4). The two main components revealed by Principal Components Analysis (PCA) explained 77.9% of the variance. Clusters A and D contained lines with extreme values for most traits, while those in B and C exhibited intermediate values (see Table S3). A close correspondence was detected with the classifications obtained using the SBP. Most lines (39) from the SBP and Core SBP were grouped into the same cluster using HCPC analysis. Changes to classifications were detected between Clusters A and B (5 lines) and Clusters C and D (7 lines). The Core-SBP also maintained a high diversity of pod color (26 green, three mottled green, one mottled yellow, six purple, and 18 yellow) and seed coat color (six black, nine brown, eight canella, eight cream, eight dark brown, four purple, three red, and eight white). Finally, estimating the Shannon and Simpson diversity indices revealed higher values in the Core-SBP than in the SBP (see Table S4). The mean values for the Shannon and Simpson diversity indices increased from 1.06 and 0.53 to 1.32 and 0.68, respectively.

The Core-SBP was phenotyped for determinate growth habit (*fin* gene) and also genotyped with 24 InDel markers found at the map locations associated with genes/QTLs controlling pod traits. The number of alleles ranged between 2 and 5, and the minor allele frequency (MAF) was between 0.02 and 0.49 (Table S5). The markers Ind_1_45.4584, Ind_1_51.6243, Ind_2_43.1499, and Ind_6_20.0131 were removed from the analysis due to a MAF < 0.02 (Table S5). Regarding pod dimensions (length and section traits), the results revealed significant associations with nine regions that were previously reported to be involved in the control of pod phenotype. These regions were tagged by seven InDels and the gene *fin* (Table 3 and Table S5). The InDel Ind_1_19.1533 was the only marker associated with cross-section traits, and this region has not previously been reported as being associated with pod dimensions. In terms of the NSP, the findings indicated an association with six regions in chromosomes Pv01, Pv04, Pv06, and Pv07. Finally, with respect to pod color, Chi-square tests revealed a significant association with two regions previously associated with pod color control (Table S6). Yellow pod color was associated with the marker Ind_2_0.8980 (Yellow & No Yellow; Χ^2^ = 14.1, *p* < 0.001), while purple pod color was shown to be associated with the marker Ind_2_48.6551 (Purple & No purple; Χ^2^ = 8.14, *p* < 0.004).

## 3. Discussion

Phenotyping often constitutes a bottleneck in diversity studies, given the resources and time required for its development. Most diversity studies are now based on DNA markers. However, phenotype is an essential component of strategies for breeding and preserving biodiversity. Here we described the phenotypic diversity in a set of snap beans collected from European gene banks, seeds companies, and working collections. Phenotyping was focused on pod traits, but included reference to growth habit, along with crop yield and adaptation. Pod phenotyping included the longitudinal and cross-sectional traits, which are closely related to their potential uses. Cultivars that are harvested to be processed require straight pods that are homogeneous in length and have round cross-sections, while for the fresh vegetable market this characteristic can be more variable [11]. The SBP encompassed wide phenotypic variation in pod size, cross-sections, and NSP. The SBP also contained a wide variation of pod colors, a characteristic associated with consumer preference and an indicator of postharvest quality [23]. The pod colors varied between green, yellow, and purple, although the purple color is not maintained during cooking. Seed size and color are also related to pod quality. Cultivars with white seed coats are commonly preferred for processing due to pigments [anthocyanins] causing an off-color in the pre-cooked product [18]. Moreover, many cultivars developed for processing have long and cylindrical seed shapes due to the selection of long and round pods. This panel contained cultivars with seeds of a wide range of shapes and sizes (see Table 1), including small, cylindrical, and white seeds (e.g., SBP028, SBP150), or large and colored seeds (e.g., SBP006; SBP040, SBP245).

In the SBP, most lines had determinate growth habits (237). Lines with indeterminate bush or prostrate growth habits were not found in this panel, indicating that many of them probably derive from breeding programs. Wild common beans all have indeterminate growth habits, and the plants flower under short day conditions. Determinacy has been adopted at higher latitudes or in cooler climates to select earlier varieties adapted to shorter growing seasons. This selection is facilitated by the linkage between the loci that control determinacy (*fin*) and the major photoperiod insensitivity gene (*ppd*) on chromosome 1 [24,25]. Therefore, it is not a surprise that most snap beans show determinate growth habits with a shortened period of pod production, leading to a more homogeneous harvest [26] and providing the opportunity for mechanical harvesting. Determinate growth habit can be an advantage for modern snap bean production, but did not always represent an advantageous trait. Indeterminate climbing growth habit has the advantage that they can be hand-harvested and have a broader window of harvest. It was common until the late 1960s and is now grown in small orchards and greenhouses. It was also observed that the determinate lines had significantly smaller pods than the indeterminate lines (e.g., PL = 12.9 cm in determinate lines and 15.8 cm in indeterminate lines; Student’s *t*-test, *t* = 7.4, *p* < 0.0001), which is likely an adaptation to avoid damage to pods by contact with the soil.

The SBP includes many lines (311), and its study, use and preservation require many resources. Its characterization also revealed putative redundant lines showing similar phenotypes, so the establishment of a core set with a minimum number of lines that represent it will facilitate its handling and assist its use and maintenance. For this purpose, phenotypic characteristics were considered, including pod length and cross-section, pod color, NSP, and seed weight and color. The proposed Core-SBP contains 54 lines (17% of the SBP) and maintains wide phenotypic variation and diversity. In fact, in terms of the four phenotyping characteristics considered, the Core-SBP had higher values than the SBP for the Shannon and Simpson diversity indices (see Table S4). Additionally, the Core-SBP included the main snap bean market classes (see Figure 2), containing Romano (e.g., SBP108), Garrafal (e.g., SBP040), Blue Lake (SBP073), and Yellow Wax (e.g., SBP090).

The genetic control of the traits used for phenotyping involved both major genes and QTLs. Many QTLs associated with the control of pod size have been located in common genomic regions of bean using bi-parental populations and diversity panels (see [16,27]). Moreover, major genes with complex epistatic interactions controlling pod color have been reported in the literature, including the genes *B* (chromosome Pv02), *V* (Pv06), *P* (Pv07), *complex C locus* (Pv08), *T* (Pv09), and *J* (Pv10) [28]. Therefore, the phenotypic characterization of pods is based on variations at many loci. The Core SBP was used to verify the involvement of some of these regions in the genetic control of pod size and color by tagging the lines with InDel markers. The 24 InDel markers analyzed were polymorphic (four with a MAF < 0.02), and 12 of them were significantly associated with pod traits. Of note were observed associations among pod dimension traits (longitudinal and section) and three InDels (Ind_2_3.6382, Ind_5_29.0512, Ind_6_18.2115) located at positions 3.61, 30.35, and 17.44 Mb of chromosomes Pv02, Pv05, and Pv06, respectively. These positions overlap regions in which QTLs related to pod dimensions in two bi-parental populations and the Spanish Diversity Panel were located by Murube et al. [27] and Garcia Fernández et al. [15]. Five genomic regions were tested for pod color and two significant associations were detected on chromosome Pv02. One at the beginning of chromosome Pv02 (0.85Mb) was a region in which the gene *y* controlling yellow pod color is located [15] and the gene *Phvul.002G006200* was proposed as a potential candidate gene [29]. The results verified the involvement of these regions and validated the use of a breeder-friendly DNA marker, namely Ind_2_0.8980. Likewise, the results showed a significant association between purple color and the InDel marker Ind_02_48.6551, located at 49.27 Mb (chromosome Pv02). Gene *B*, which regulates the production of anthocyanin precursors in the seed coat color pathway, above the level of dihydrokaempferol formation, has been mapped to this position [30]. It was mapped to the telomeric region of chromosome Pv02 and was previously noted to be involved in the control of pod color [31,32]. The results confirmed the relevant role of the region containing gene *B* in producing a purple pod color. Regarding pod dimensions, the results provided by the association analyses allowed us to define the associated regions in chromosomes Pv01, Pv02, Pv05, Pv06, and Pv07 as being very relevant to the genetic control of pod phenotype and potentially useful in plant breeding. The absence of significant associations with previously reported regions may be due to the size and composition of the Core-SBP. Note that many QTLs were revealed by the analysis of specific biparental populations, in which specific polymorphisms between parents were seen to segregate.

The Core-SBP established in this study is a first attempt to classify snap bean cultivars based on morphological pod traits and constitutes a valuable source of traits for breeding programs, since it gathers a diverse range of pod morphologies. Likewise, the lines included in the Core SBP are a source of genotypes to use in investigating the genetic control of interesting traits for snap bean production by analyzing bi-parent populations, multi-parent populations, or association panels [33]. Finally, additional phenotyping and genotyping of the SBP may lead to validation of changes in the composition of the Core-SBP in order to better represent snap bean diversity.

## 4. Materials and Methods

### 4.1. Plant Material

A total of 311 snap bean accessions were gathered from European gene banks, working collections, and seed companies. The set of accessions included genotypes classified as landraces, old and elite cultivars, as well as 39 snap bean lines in common with the Spanish Diversity Panel [17]. One homozygous line per accession was obtained by self-pollination in a greenhouse of one plant derived from each accession to constitute the Snap Bean Panel (SBP, Table S7).

#### 4.1.1. Phenotyping

The SBP was evaluated at Villaviciosa, Spain (43°2901 N, 5°2611 W; elevation 6.5 m). The lines were characterized in the greenhouse during 2018 (23 July 2018 to 30 October 2018) and in the field during 2020 (18 May 2020 to 30 September 2020). A randomized design with one plot per line was used for both trials. A plot included 8–10 plants per line distributed in 1 m. The seeds were germinated in trays containing peat and then transplanted to ensure the homogeneity of the crop. The field crops were mulched with plastic to control weeds, and organic farming management practices were followed to ensure adequate plant growth and development. The greenhouse and field crops were developed on loam soil (pH = 7.4 and 2.43% organic matter; see Figure S4).

Pod phenotypic diversity was recorded by phenotyping four types of traits: pod morphological characters, fresh pod color, main seed coat color, and plant growth habit. Pod morphology included 12 dimension traits (see Table 1), NSP, and 25-seed weight. Pod dimensions were measured longitudinally (PLP, PLA, PLW, PL, PLC, PL/PLC) and in the cross-section (PSP, PSA, PSW, PSH, PSH/PSW, PSC) for 10 fresh pods (at the beginning of developmental stage R8) per line with the help of Tomato Analyzer software [34]. The NSP was manually recorded from 10 dry pods, and the 25-seed weight was evaluated four times per line in dry seeds (14% water content). Fresh pod color and main seed coat color were qualitatively recorded in five (green, yellow, mottled green, mottled yellow, and purple) and nine (white, yellow, cream, canella, brown, dark brown, red, purple, and black) classes, respectively. Finally, the growth habit was recorded when the plants started flowering, by considering the four main classes [35]: (i) determinate bush; (ii) indeterminate bush; (iii) indeterminate prostrate, and (iv) indeterminate climbing.

#### 4.1.2. Statistical Analysis

The phenotypic variation in the quantitative morphological traits was visualized by frequency distributions generated by ggplot2 [36]. Descriptive statistical analyses of the phenotypic data were conducted in R [37] using the package Rcmdr [38]. Means derived from the different evaluations were adjusted using least-squares means with the help of the package LSmeans [39], and normality was analyzed by Shapiro-Wilk tests. Correlation coefficients among the traits were also investigated using the package corrplot [40].

An HCPC analysis was carried out to identify the main clusters using 14 morphological characteristics of pods. The HCPC approach allowed us to combine the three standard methods used in multivariate data analyses: PCA, hierarchical clustering, and partitioning clustering. This analysis was performed in the R platform using the packages ggplot2, FactoMiner, and FactoExtra [41]. The differences among the clusters established using the HCPC were investigated by mean ANOVA followed by a post-hoc Tukey test in Rcmdr [38].

#### 4.1.3. Establishment of a Core Set for the SBP

A hierarchical procedure was followed to establish a subset of lines (Core-SBP) that represented most of the phenotypic diversity gathered in the SBP [42]. The lines were first grouped according to the results provided by HCPC from the 14 quantitative morphological traits, and within each group the lines were classified according to the pod color and then the main seed coat color. One line per established phenotypic class (HCPC cluster, pod color, and seed color) was randomly selected to represent it and become part of the Core-SBP. The Shannon-Wiener diversity index (H’; [43]) and Simpson diversity index (1-D; [44]) were calculated to compare the differences between the SBP and Core-SBP for each trait.

#### 4.1.4. Core SBP Phenotyping and Genotyping

Candidate lines selected to be included in the Core-SBP were phenotyped in the field during summer 2021 using the same set of descriptors. A randomized design with three plots per line was used. A plot included 8–10 plants per line distributed in 1 m. The crop was developed during the period 7 May 2021 to 16 September 2021 at Villaviciosa, Spain, and organic farming management practices were followed to ensure adequate plant growth and development.

The Core-SBP was used to investigate the variation at breeder-friendly DNA markers that tagged genomic regions in which 19 QTLs involved in the control of pod morphology were previously reported [15,27]. The 24 InDel markers described by Moghaddam et al. [45] were chosen to tag these regions. At least one polymorphic IndDel per genomic region was selected by considering its physical position in the bean genome V2.1 (https://phytozome-next.jgi.doe.gov/, accessed on 15 November 2021). Fresh young trifoliate leaves from each line were collected, frozen, and homogenized into a fine powder to isolate the genomic DNA using the SILEX method [46].

Significant associations between markers and pod traits were investigated using Student’s *t*-test (marker with two alleles) or ANOVA (markers with more than two alleles). To detect the association between qualitative traits and InDel markers, Chi-contingency tests were used. Significant differences (*p* < 0.05) among the alleles of the markers indicated the involvement of the tagged region in the control of the trait. Statistical analysis was carried out using package Rcmdr in R [37,38].

## Figures and Tables

**Table 1:** List of the 12 pod traits analyzed using the software Tomato Analyzer. The code assigned to each character is indicated in parentheses. The green pods were collected at the beginning of the R8 stage, pods developed, and seeds grew.

Characters	Traits	Unit	Description
Pod section (PS) ^1^	Pod Section Perimeter (PSP)	cm	The perimeter of section, measure of 10 randomly chosen green pods
	Pod Section Area (PSA)	cm^2^	Area of section, measure of 10 randomly chosen green pods
	Pod Section Width (PSW)	cm	Width of section, measure of 10 randomly chosen green pods, taken perpendicular to suture
	Pod Section Height (PSH)	cm	Height of section, measure of 10 randomly chosen green pods, taken parallel to the suture
	Pod Section index (PSHPSW)		PSH/PSW ratio
	Pod Section circular (PSC)		Fit of a circular shape of the section
Pod length (PL)	Pod Length Perimeter (PLP)	cm	The perimeter of longitudinal section, measure of 10 randomly chosen green pods
	Pod Length Area (PLA)	cm^2^	Area of longitudinal section, measure of 10 randomly chosen green pods
	Pod Length Width (PLW)	cm	Width of transversal section, measure of 10 randomly chosen green pods at the mid-length
	Pod Length (PL)	cm	Length of measure of 10 randomly chosen green pods
	Pod Length Curved (PLC)	cm	Length of measure along a curved line through the pod of 10 randomly chosen green pods
	Pod Length index (PLPLC)		Index for the level of curvature measure as PL/PLC ratio

^1^ to measure the pod cross-section characters, the green pods were cut between the position of the second and third seed analysed.

**Table 2:** Means, interval variation (Min, Max), and standard errors (SE) for the 14 quantitative pod traits recorded in the Snap Bean Panel (SBP) and core set established from this SBP (Core-SBP).

SBP							Core-SBP		
Traits	Unit	Mean	Min	Max	SE	Mean	Min	Max	SE
PL	cm	13.61	7.31	25.86	0.18	11.93	7.07	19.01	0.39
PLA	cm^2^	13.48	4.58	50.69	0.45	16.69	6.48	35.66	1.05
PLC	cm	13.36	7.32	25.66	0.17	14.31	9.96	22.05	0.40
PLP	cm	30.19	15.95	57.28	0.41	32.44	21.63	50.24	0.95
PLPLC		1.02	0.84	1.09	0.00	0.84	0.52	0.97	0.02
PLW	cm	1.04	0.49	2.29	0.02	1.21	0.58	2.01	0.06
PSA	cm^2^	0.64	0.23	1.30	0.01	0.68	0.33	1.18	0.03
PSC		0.11	0.02	0.35	0.01	0.18	0.03	0.34	0.01
PSH	cm	1.08	0.56	2.18	0.02	1.23	0.66	1.91	0.05
PSHPSW		1.51	0.88	3.59	0.04	1.88	0.89	3.45	0.09
PSP	cm	3.21	1.83	5.32	0.04	3.39	2.17	4.80	0.09
PSW	cm	0.74	0.50	1.04	0.01	0.68	0.44	0.85	0.01
NSP	seeds	5.87	3.70	8.75	0.05	5.78	4.1	8.12	0.12
25 Seedweight	g	9.18	1.92	22.60	0.23	11.65	3.355	21.27	0.54

**Table 3:** Significant associations marker-trait detected in the Core-SBP among marker loci tagging QTL/genes for pod characters recorded in the 2021 field trial. MAF, Minor allele frequency. PL, pod length traits (PL, PLA, PLC, PLPLC). PS, pod cross-section traits (PSA, PSC, PLW, PSH, PSHPSW, PSP). NSP, number of seeds per pod; Color, fresh pod color.

Marker Loci	Physical Position ^(1)^		Associated Pod Traits	N Alleles	MAF	Pod Length	Pod Section	NSP	Pod Color
Ind_1_19.1533	Pv01	16039072	NSP	4	0.04	ns	sa	sa	
Ind_1_38.7943	Pv01	38145306	PL, PS	2	0.13	ns	ns	ns	
Gene Fin	Pv01	44857680	PL, PS	2	0.38	sa	sa	sa	
Ind_1_47.2870	Pv01	46596666	PL	3	0.04	ns	ns	sa	
Ind_2_0.8980	Pv02	855516	Color	3	0.15				sa
Ind_2_2.4495	Pv02	2405590	Color	3	0.02				ns
Ind_2_3.6382	Pv02	3615508	PL, PS	3	0.20	sa	sa	ns	
Ind_2_28.4405	Pv02	29688140	PL	2	0.49	sa	sa		
Ind_2_48.6551	Pv02	49273667	NSP, PL, PS, Color	2	0.31	ns	ns	ns	sa
Ind_3_48.9580	Pv03	50111660	PL, NSP	2	0.08	ns	ns	ns	
Ind_4_39.8831	Pv04	41925005	PL, PS	3	0.18	sa	sa	sa	
Ind_4_42.2659	Pv04	44371105	PL, PS	3	0.07	ns	ns	ns	
Ind_5_29.0512	Pv05	30358343	PL	3	0.22	sa	sa		
Ind_5_39.3321	Pv05	39571081	PS	3	0.11	sa	sa		
Ind_5_39.8141	Pv05	40039649	PS	*5*	*0.04*	sa	sa		
Ind_6_18.2115	Pv06	17449107	NSP, PL, PS,	2	0.29	sa	sa	sa	
Ind_7_6.6340	Pv07	6784415	NSP, PS	2	0.22	sa	sa	sa	
Ind_8_57.1490	Pv08	60556455	PS, Color	3	0.07	ns	ns	ns	ns
Ind_8_57.3095	Pv08	60743579	PS, Color	2	0.11	ns	ns	ns	ns
Ind_11_2.3017	Pv11	2471919	PL	5	0.09	ns	ns	ns	
Ind_11_4.2292	Pv11	4391403	PS	2	0.29	ns	ns	ns	

ns, not significant association (*p* > 0.05), sa, significant association (see also Tables S6 and S7). ^(1)^ *P. vulgaris* genome V2.1.

## Data Availability

Not applicable.

## Supplementary Materials

The following supporting information can be downloaded at: https://www.mdpi.com/article/10.3390/plants11050577/s1, Table S1: Mean per cluster revealed by HCPC analysis for the evaluated pod traits in the SBP (311 lines), Table S2: Phenotypic classes considered from HCPC (cluster A, B, C, and D), pod color (green, yellow, mottled green, mottled yellow, and purple), and main seed color (white, yellow, cream, canella, brown, dark brown, red, purple and black), Table S3: Mean per cluster revealed by HCPC analysis for the evaluated pod traits in the Core- SBP (51 lines characterized). Means in each row followed by the same letters indicate non-significant differences after applying the Tukey test, Table S4: Estimation of the diversity index of Shannon (H’) and Simpson (1-D) in the Snap Bean Panel (SBP) and the Core-SBP, Table S5: Significant associations revealed by T-Student test (marker loci with two alleles) and Analysis of variance (more than two alleles) for the quantitative pod traits and the marker loci analyzed in the Core-SBP. *ns*, not significant differences (*p* > 0.05), Table S6: Significant associations revealed by chi contingency tests for the pod color and marker loci located in the position in which previously were reported genes for pod color. Table S7: List of the 311 snap bean lines included in snap bean panel (SBP). Imagens of pods can be checked at https://zenodo.org/record/5557139#.YdMW0VmCGUk, Figure S1: Histograms showing the distributions (mean of two environments data adjusted with LSmeans) for the 14 quantitative pod traits assessed in the Snap Diversity Panel. Kolmogorov-Smirnov normality test (K-S), Figure S2: Corrplot showing the Spearman correlation among the 14 quantitative pod traits evaluated. Non-significant correlations (α = 0.05) are indicated with ‘X’, Figure S3: Biplots showing the results of the Hierarchical Clustering on Principal Components analysis from 14 evaluated quantitative pod traits in the Core-SBP. Ellipses representing the clusters were drawn considering a confidence interval > 0.8. Figure S4. Temperature (daily average in °C) and rainfall (mm) were recorded in the field crops during 2020 and 2021. The crops were carried out between May and September.
