# Supplementary material for: A Core Set of Snap Bean Genotypes Established by Phenotyping a Large Panel Collected in Europe
Source: Plants (Basel). 2022 Feb 22;11(5):577. doi: 10.3390/plants11050577 (PMC8912712; doi:10.3390/plants11050577)
Supplement: Supplementary file 1 [file plants-11-00577-s001.zip › plants-1600751-supplementary.pdf]

A Core Set of Snap Bean Genotypes Established by Phenotyping a Large Panel Collected in Europe

Carmen García-Fernández [1], Maria Jurado [1], Ana Campa [1], Creola Brezeanu [2], Valérie Geffroy [3], Elena Bitocchi [4], Roberto Papa [4] and Juan Jose Ferreira [1,*]

[1] Plant Genetic Group, Regional Service for Agrofood Research and Development (SERIDA), 33300, Villaviciosa, Asturias, Spain; cgarcia@serida.org (C. G.-F.), mjurado@serida.org (M. J.) and acampa@serida.org (A. C.)
[2] Stațiunea de Cercetare Dezvoltare pentru Legumicultură, Bacău, Romania. (SCDL Bacău); creola.brezeanu@legumebac.ro
[3] Université Paris-Saclay, CNRS, INRAE, Univ Evry, Institute of Plant Sciences Paris-Saclay (IPS2), 91405 Orsay, France; valerie.geffroy@universite-paris-saclay.fr
[4] Department of Agricultural, Food and Environmental Sciences, Marche Polytechnic University, Via Brecce Bianche, 60131 Ancona, Italy
* Correspondence: jjferreira@serida.org

SUPPLEMENTARY MATERIALS

Table S1. Mean per cluster revealed by HCPC analysis for the evaluated pod traits in the SBP (311 lines). In each row. means followed by the same letters indicate non-significant differences after applying the Tukey test.

Trait	A		B		C		D	
**PL**	11.50	a	14.68	b	13.42	c	21.57	d
**PLA**	8.00	a	12.46	b	18.73	c	37.06	d
**PLC**	11.26	a	14.26	ab	13.46	ab	21.31	c
**PLP**	25.23	a	32.59	b	30.09	c	48.28	d
**PLPLC**	1.01	bc	1.03	c	0.97	a	1.01	b
**PLW**	0.77	a	0.92	b	1.55	c	1.81	d
**PSA**	0.47	a	0.63	b	0.86	c	1.03	d
**PSC**	0.06	a	0.07	a	0.24	b	0.28	c
**PSH**	0.83	a	0.97	b	1.53	c	1.81	d
**PSHPSW**	1.19	a	1.20	a	2.32	b	2.72	c
**PSP**	2.66	a	3.13	b	4.00	c	4.56	d
**PSW**	0.70	a	0.82	b	0.67	a	0.68	a
**NSP**	5.83	ab	5.94	b	5.43	a	7.14	c
**25-Seed weight**	6.04	a	9.21	b	13.60	c	14.51	c

Table S2. Phenotypic classes considered from HCPC (cluster A, B, C, and D), pod color (green, yellow, mottled green, mottled yellow, and purple), and main seed color (white, yellow, cream, canella, brown, dark brown, red, purple and black). The randomly selected line in each class is also indicated as well as the presence in the Spanish Diversity Panel (SDP). *, Data non-available from field evaluation during 2021.

**Class**	**Cluster**	**Pod Color**	**Seed color**	**N. lines**	**Selected Line**	**SDP**	**Growth habit**
1	**A**	GREEN	BLACK	8	**SBP367**		Det
2	**A**	GREEN	BROWN	2	**SBP091**		Det
3	**A**	GREEN	DARK BROWN	4	**SBP073**		Det
4	**A**	GREEN	CANELLA	2	**SBP085**		Det
5	**A**	GREEN	PURPLE	1	**SBP227**		Det
6	**A**	GREEN	RED	1	**SBP374**		Det
7	**A**	GREEN	WHITE	73	**SBP028**	**SDP273**	Det
8	**A**	PURPLE	DARK BROWN	1	**SBP051**		Det
9	**A**	PURPLE	CANELLA	1	**SBP029**	**SDP206**	Det
10	**A**	PURPLE	CREAM	1	**SBP042**		Det
11	**A**	YELLOW	BLACK	5	**SBP024**	**SDP011**	Det
12	**A**	YELLOW	BROWN	3	**SBP080**	*****	Det
13	**A**	YELLOW	DARK BROWN	2	**SBP249**		Det
14	**A**	YELLOW	CREAM	1	**SBP103**		Det
15	**A**	YELLOW	WHITE	16	**SBP150**		Det
16	**B**	GREEN	BLACK	20	**SBP014**	**SDP271**	Ind
17	**B**	GREEN	BROWN	4	**SBP245**		Det
18	**B**	GREEN	DARK BROWN	18	**SBP116**		Ind
19	**B**	GREEN	CANELLA	3	**SBP077**		Det
20	**B**	GREEN	CREAM	7	**SBP113**		Det
21	**B**	GREEN	PURPLE	5	**SBP350**		Det
22	**B**	GREEN	WHITE	23	**SBP032**	**SDP230**	Det
23	**B**	MOTTLED GREEN	CREAM	6	**SBP179**		Det

24	**B**	PURPLE	CANELLA	2	**SBP030**	**SDP205**	Ind
25	**B**	YELLOW	BLACK	7	**SBP265**	*****	Det
26	**B**	YELLOW	BROWN	1	**SBP090**		Det
27	**B**	YELLOW	DARK BROWN	2	**SBP270**		Det
28	**B**	YELLOW	WHITE	11	**SBP054**		Ind
29	**C**	GREEN	BLACK	5	**SBP333**		Ind
30	**C**	GREEN	BROWN	2	**SBP318**		Det
31	**C**	GREEN	DARK BROWN	4	**SBP307**		Ind
32	**C**	GREEN	CANELLA	4	**SBP026**	**SDP210**	Det
33	**C**	GREEN	CREAM	2	**SBP344**		Det
34	**C**	GREEN	PURPLE	2	**SBP040**	**SDP143**	Det
35	**C**	GREEN	RED	2	**SBP082**		Ind
36	**C**	GREEN MOTTLED	WHITE	8	**SBP005**	**SDP204**	Det
37	**C**	GREEN MOTTLED	CREAM	3	**SBP279**		Det
38	**C**	YELLOW	BROWN	1	**SBP007**	**SDP207**	Det
39	**C**	PURPLE	CREAM	1	**SBP053**		Ind
40	**C**	YELLOW	BLACK	8	**SBP308**		Ind
41	**C**	YELLOW	BROWN	8	**SBP336**		Det
42	**C**	YELLOW	DARK BROWN	7	**SBP037**	**SDP035**	Ind
43	**C**	YELLOW	CANELLA	1	**SBP311**		Ind
44	**C**	YELLOW	PURPLE	2	**SBP093**		Ind
45	**C**	YELLOW	WHITE	1	**SBP326**	*****	Det
46	**D**	GREEN	DARK BROWN	3	**SBP101**		Ind
47	**D**	GREEN	CANELLA	1	**SBP010**	**SDP245**	Ind
48	**D**	GREEN	RED	1	**SBP108**		Det
49	**D**	GREEN MOTTLED	WHITE	7	**SBP015**	**SDP203**	Ind
50	**D**	GREEN	BROWN	1	**SBP006**	**SDP211**	Ind
51	**D**	PURPLE	CREAM	1	**SBP096**		Ind
52	**D**	YELLOW	BROWN	2	**SBP092**		Ind
53	**D**	YELLOW	CANELLA	1	**SBP041**	**SDP156**	Ind

54	**D**	YELLOW	WHITE	2	**SBP008**	**SDP270**	Ind

Table S3. Mean per cluster revealed by HCPC analysis for the evaluated pod traits in the Core- SBP (51 lines characterized). Means in each row followed by the same letters indicate non-significant differences after applying the Tukey test.

Trait	**A**		**B**		**C**		**D**	
**PL**	9.66	a	11.58	a	11.66	a	18.90	b
**PLA**	9.33	a	14.35	b	15.91	b	26.83	c
**PLC**	12.05	a	16.24	b	13.13	a	18.06	b
**PLP**	27.14	a	37.95	b	29.44	a	38.67	b
**PLPLC**	0.81	ab	0.72	a	0.89	b	0.88	b
**PLW**	0.82	a	0.88	a	1.33	b	1.69	c
**PSA**	0.51	a	0.60	ab	0.67	b	0.93	c
**PSC**	0.09	a	0.08	a	0.24	b	0.27	b
**PSH**	0.88	a	0.93	a	1.34	b	1.66	c
**PSHPSW**	1.27	s	1.20	s	2.26	b	2.54	b
**PSP**	2.74	a	3.00	a	3.48	b	4.22	c
**PSW**	0.70	bc	0.78	c	0.60	a	0.66	ab
**NSP**	5.60	a	6.67	b	5.25	a	6.09	ab
**25-seed weight**	8.78	a	9.63	ab	12.28	bc	15.26	c

Table S4. Estimation of the diversity index of Shannon (H′) and Simpson (1-D) in the Snap Bean Panel (SBP) and the Core-SBP.

		SBP			Core_SBP		
**Trait**	**Classes**	n	**H´**	**1-D**	**n**	**H**	**1-D**
**Pod dimensions**	Cluster A	121	**1.22**	**0.69**	17	**1.3**	**0.71**
	Cluster B	109			6		
	Cluster C	62			13		
	Cluster D	19			15		
**Pod color**	Green	212	**0.87**	**0.49**	26	**1.27**	**0.67**
	Yellow	80			18		
	Mottled green	17			3		
	Mottled yellow	1			3		
	Purple	7			6		
**Seed coat color**	White	141	**1.63**	**0.74**	8	**2.02**	**0.863**
	Yellow	0			0		
	Cream	22			8		
	Canella	15			8		
	Brown	25			9		
	Dark brown	41			8		
	Red	4			3		
	Purple	10			4		
	Black	53			6		
**Growth habit**	Determinate	237	**0.54**	**0.21**	34	**0.65**	**0.47**
	Indeterminate	74			20		
Mean			1.06	0.53		1.32	0.68

Table S5. Significant associations were revealed by the T-Student test (marker loci with two alleles) and Analysis of variance (more than two alleles) for the quantitative pod traits and the marker loci analyzed in the Core-SBP. *ns*, not significant differences (p>0.05).

			**Pod length**					**Pod section**								
Chr	**Marker loci**	N. Alleles	PLA	PL	PLC	PLP	PL/ PLC	PLW	PSA	PSC	PSH	PSH/PSW	PSP	PSW	NSP	**25-seed weight**
PV01	***Ind_1_19.1533***	4	ns	ns	ns	ns	ns	ns	0.003	0.012	0.003	0.027	0.003	0.002	0.016	0.002
PV01	***Ind_1_38.7943***	2	ns	ns	ns	ns	ns	ns	ns	ns	ns	ns	ns	ns	ns	ns
PV01	***Ind_1_45.4584***	2					MAF<0.02									
PV01	***Gene Fin/fin***	2	0.001	0.009	0.009	0.012	ns	0.002	0.01	0.007	0.001	0.004	1E-05	0.047	0.001	ns
PV01	***Ind_1_47.2870***	3	ns	ns	ns	ns	ns	ns	ns	ns	ns	ns	ns	ns	0.001	ns
PV01	***Ind_1_51.6243***	2					MAF<0.02									
PV02	***Ind_2_0.8980***	3	ns	ns	ns	ns	ns	ns	ns	ns	ns	ns	ns	ns	ns	ns
PV02	***Ind_2_2.4495***	3	ns	ns	ns	ns	ns	ns	ns	ns	ns	ns	ns	ns	ns	ns
PV02	***Ind_2_3.6382***	3	0.0001	ns	ns	ns	ns	0.0001	0.0001	0.0001	0.0001	0.0001	0.0001	ns	ns	0.0001
PV02	***Ind_2_28.4405***	2	ns	ns	ns	ns	ns	ns	ns	0.004	0.013	0.0001	0.04	0.01	ns	ns
PV02	***Ind_2_43.1499***	2					MAF<0.02									
PV02	***Ind_2_48.6551***	2	ns	ns	ns	ns	ns	ns	ns	ns	ns	ns	ns	ns	ns	ns
PV03	***Ind_3_48.9580***	2	ns	ns	ns	ns	ns	ns	ns	ns	ns	ns	ns	ns	ns	ns
PV04	***Ind_4_39.8831***	3	ns	ns	ns	ns	ns	ns	ns	ns	ns	ns	ns	ns	ns	ns
PV04	***Ind_4_42.2659***	3	ns	ns	ns	ns	ns	ns	ns	ns	ns	ns	ns	ns	ns	ns
PV05	***Ind_5_29.0512***	3	0.002	0.014	0.04	0.04	ns	0.009	0.005	0.043	0.009	ns	0.006	ns	0.036	ns
PV05	***Ind_5_39.3321***	3	ns	0.018	0.014	0.032	ns	ns	ns	ns	ns	ns	ns	ns	ns	ns
PV05	***Ind_5_39.8141***	5	0.013	ns	0.017	0.011	ns	0.046	ns	0.048	0.032	ns	ns	ns	ns	0.034
PV06	***Ind_6_18.2115***	2	0.01	0.005	0.03	0.04	ns	0.01	0.0001	0.001	0.0001	0.0001	0.0001	ns	0.01	ns
PV06	***Ind_6_20.0131***	2					MAF<0.02									
PV07	***Ind_7_6.6340***	2	0.031	0.037	0.011	0.011	0.012	ns	ns	ns	ns	ns	ns	ns	ns	ns
PV08	***Ind_8_57.1490***	3	ns	ns	ns	ns	ns	ns	ns	ns	ns	ns	ns	ns	ns	ns
PV08	***Ind_8_57.3095***	2	ns	ns	ns	ns	ns	ns	ns	ns	ns	ns	ns	ns	ns	ns
PV11	***Ind_11_2.3017***	5	ns	ns	ns	ns	ns	ns	ns	ns	ns	ns	ns	ns	ns	ns

PV11	***Ind_11_4.2292***	2	ns	ns	ns	ns	ns	ns	ns	ns	ns	ns	ns	ns	ns	ns

Table S6. Significant associations were revealed by chi contingency tests for the pod color and marker loci located in the position in which previously were reported genes for pod color.

			**Green&No Green**		**Yellow & No Yellow**		**Purple& No Purple**	
**Chr**	**Marker loci**	**N. alleles**	**X^2**	***p-value***	**X^2**	***p-value***	**X^2**	***p-value***
Pv02	*Ind_2_0.8980*	3	3.883	0.144	14.193	0.001	1.074	0.584
Pv02	*Ind_2_2.4495*	3	2.879	0.237	1.281	0.527	1.876	0.392
Pv02	*Ind_2_48.6551*	2	0.928	0.335	0.839	0.360	8.137	0.004
Pv08	*Ind_8_57.1490*	3	2.233	0.327	1.468	0.480	2.671	0.263
Pv08	*Ind_8_57.3095*	2	1.968	0.161	3.195	0.074	0.021	0.884

Table S7 List of the 311 snap bean lines included in snap bean panel (SBP). Images of pods can be checked at https://zenodo.org/record/5557139#.YdMW0VmCGUk.

Line	Donor (1)	Donor Code/ other code	Cultivar name	DOI NUMBER
**SBP002**	SERIDA	**SDP224**	CONTENDER	**10.18730/SC0M7**
**SBP003**	SERIDA	**SDP251**	MANTECA DE LOS MERCADOS	**10.18730/SC0N8**
**SBP004**	SERIDA	**SDP247**	ILERDA	**10.18730/SC0P9**
**SBP005**	SERIDA	**SDP204**	BINA	**10.18730/SC0QA**
**SBP006**	SERIDA	**SDP211**	BUENOS AIRES ROJA	**10.18730/SC0RB**
**SBP007**	SERIDA	**SDP207**	BOCA DE DRAGON	**10.18730/SBCAQ**
**SBP008**	SERIDA	**SDP270**	PATXI	**10.18730/SC0SC**
**SBP010**	SERIDA	**SDP245**	GARRAFAL ORO	**10.18730/SBC1E**
**SBP011**	SERIDA	**SDP259**	MUSICA	**10.18730/SBC0D**
**SBP012**	SERIDA	**SDP244**	GOLDMARIE	**10.18730/SC0TD**
**SBP013**	SERIDA	**SDP255**	MARCONI	**10.18730/SC0VE**
**SBP014**	SERIDA	**SDP271**	PERFECCION NEGRA POLO	**10.18730/SBC8N**
**SBP015**	SERIDA	**SDP203**	BILMA	**10.18730/SC0WF**
**SBP016**	SERIDA	**SDP241**	GARRAFAL ENANA	**10.18730/SC0XG**
**SBP017**	SERIDA	**SDP265**	NASSAU	**10.18730/SC0YH**
**SBP018**	SERIDA	**SDP006**	AMARILLA CAPITANO	**10.18730/SC0ZJ**
**SBP019**	SERIDA	**SDP288**	SUPERBA	**10.18730/SC10K**
**SBP020**	SERIDA	**SDP007**	AMETHYST	**10.18730/SC11M**
**SBP021**	SERIDA	**SDP268**	NURIA	**10.18730/SC12N**
**SBP022**	SERIDA	**SDP258**	MONEL	**10.18730/SC13P**
**SBP023**	SERIDA	**SDP232**	FIN DE BAGNOLS	**10.18730/SC14Q**
**SBP024**	SERIDA	**SDP011**	BEURRE DE ROCQUENCOURT	**10.18730/SBC5J**
**SBP025**	SERIDA	**SDP242**	GLOIRE DE SAUMUR	**10.18730/SC15R**
**SBP026**	SERIDA	**SDP210**	BROWN DUTCH	**10.18730/SBC6K**
**SBP027**	SERIDA	**SDP229**	DUBLETTE	**10.18730/SC16S**
**SBP028**	SERIDA	**SDP273**	PINZESSA	**10.18730/SC17T**
**SBP029**	SERIDA	**SDP206**	BLUEVETTA	**10.18730/SC18V**
**SBP030**	SERIDA	**SDP205**	BLAUHILDE	**10.18730/SC19W**
**SBP031**	SERIDA	**SDP266**	NECKARGOLD	**10.18730/SC1AX**
**SBP032**	SERIDA	**SDP230**	DUPLIKA	**10.18730/SC1BY**
**SBP033**	SERIDA	**SDP243**	GOLDEN TEEPEE	**10.18730/SC1CZ**
**SBP034**	SERIDA	**SDP012**		**10.18730/SC1D***
**SBP035**	SERIDA	**SDP172**		**10.18730/SC1E~**
**SBP036**	SERIDA	**SDP107**		**10.18730/SC1F$**
**SBP037**	SERIDA	**SDP035**		**10.18730/SC1G=**

**SBP038**	SERIDA	**SDP126**		**10.18730/SC1HU**
**SBP039**	SERIDA	**SDP191**		**10.18730/SC1J0**
**SBP040**	SERIDA	**SDP143**		**10.18730/SC1K1**
**SBP041**	SERIDA	**SDP156**		**10.18730/SC1M2**
**SBP042**	SERIDA	S326	ROYAL BURGUNDY	**10.18730/SC1N3**
**SBP043**	SERIDA	S327	CUPIDON	**10.18730/SC1P4**
**SBP044**	SERIDA	S328	LAZY HOUSEWIFE	**10.18730/SC1Q5**
**SBP045**	SERIDA	S329	AIDA GOLD	**10.18730/SC1R6**
**SBP046**	SERIDA	S330	AQUILON	**10.18730/SC1S7**
**SBP048**	SERIDA	S332	CHEROKEE TRAIL OF TEARS	**10.18730/SC1T8**
**SBP049**	SERIDA	S333	HILADORA	**10.18730/SC1V9**
**SBP051**	SERIDA	S335	QUEEN	**10.18730/SC1WA**
**SBP053**	SERIDA	S337	VIOLETTE	**10.18730/SC1XB**
**SBP054**	SERIDA	S338	NECKARKONIGIN	**10.18730/SC1YC**
**SBP056**	SERIDA	S341	SPEEDY	**10.18730/SC1ZD**
**SBP057**	SERIDA	S342	NOMAD	**10.18730/SC20E**
**SBP058**	SERIDA	S343	SONESTA	**10.18730/SC21F**
**SBP059**	SERIDA	S344	CONCADOR	**10.18730/SC22G**
**SBP060**	SERIDA	S345	DULCINA	**10.18730/SC23H**
**SBP061**	SERIDA	S346	OPERA	**10.18730/SC24J**
**SBP062**	SERIDA	S348	MASCOTTE	**10.18730/SC25K**
**SBP063**	SERIDA	S349	SPENDIDO	**10.18730/SC26M**
**SBP064**	SERIDA	S350	FASOLD	**10.18730/SC27N**
**SBP065**	SERIDA	S351	THE PRINCE	**10.18730/SC28P**
**SBP066**	SERIDA	S352	SAFARI	**10.18730/SC29Q**
**SBP067**	SERIDA	S353	CASTANDEL	**10.18730/SC2AR**
**SBP068**	SERIDA	S355	MAXI	**10.18730/SC2BS**
**SBP070**	UNIVPM	ECa052		**10.18730/H7QGQ**
**SBP071**	UNIVPM	Ece003		**10.18730/H7XW5**
**SBP072**	UNIVPM	Ece031		**10.18730/H7YHT**
**SBP073**	UNIVPM	Ece204		**10.18730/H81C6**
**SBP076**	UNIVPM	Ece210		**10.18730/H81JC**
**SBP077**	UNIVPM	Ece232		**10.18730/H828$**
**SBP078**	UNIVPM	Ece237		**10.18730/H82D2**
**SBP079**	UNIVPM	Ece238		**10.18730/H82E3**
**SBP080**	UNIVPM	ECa002		**10.18730/H7P2E**
**SBP081**	UNIVPM	ECa011		**10.18730/H7PAP**
**SBP082**	UNIVPM	ECa019		**10.18730/H7PJY**
**SBP083**	UNIVPM	ECa046	MIDAS	**10.18730/H7QCK**
**SBP085**	UNIVPM	ECa183		**10.18730/H7VG3**

**SBP086**	UNIVPM	ECe002		**10.18730/H7XV4**
**SBP087**	UNIVPM	ECe006		**10.18730/H7XY7**
**SBP089**	UNIVPM	ECe019		**10.18730/H7Y8H**
**SBP090**	UNIVPM	ECe026		**10.18730/H7YEQ**
**SBP091**	UNIVPM	ECe033		**10.18730/H7YKW**
**SBP092**	UNIVPM	ECe038		**10.18730/H7YR~**
**SBP093**	UNIVPM	ECe039		**10.18730/H7YS$**
**SBP094**	UNIVPM	ECe055		**10.18730/H7YY2**
**SBP095**	UNIVPM	ECe080		**10.18730/H7Z6A**
**SBP096**	UNIVPM	ECe100		**10.18730/H7ZKQ**
**SBP097**	UNIVPM	ECe139		**10.18730/H8043**
**SBP098**	UNIVPM	ECe150		**10.18730/H80CB**
**SBP099**	UNIVPM	ECe151		**10.18730/H80DC**
**SBP100**	UNIVPM	ECe173		**10.18730/H80YX**
**SBP101**	UNIVPM	ECe174		**10.18730/H80ZY**
**SBP103**	UNIVPM	ECe200		**10.18730/H8182**
**SBP104**	UNIVPM	ECe207		**10.18730/H81F9**
**SBP105**	UNIVPM	ECe215		**10.18730/H81QH**
**SBP106**	UNIVPM	ECe221		**10.18730/H81XQ**
**SBP108**	UNIVPM	ECe229		**10.18730/H825Z**
**SBP109**	UNIVPM	ECe230		**10.18730/H826***
**SBP110**	UNIVPM	ECe233		**10.18730/H829=**
**SBP112**	UNIVPM	ECe240		**10.18730/H82G5**
**SBP113**	UNIVPM	ECe241		**10.18730/H82H6**
**SBP114**	UNIVPM	ECe244		**10.18730/H82M9**
**SBP115**	UNIVPM	ECe245		**10.18730/H82NA**
**SBP116**	UNIVPM	ECe264		**10.18730/H838X**
**SBP117**	UNIVPM	ECe279		**10.18730/H83Q7**
**SBP118**	UNIVPM	ECe280		**10.18730/H83R8**
**SBP120**	UNIVPM	ECe295		**10.18730/H847Q**
**SBP121**	UNIVPM	ECe302		**10.18730/H84EY**
**SBP122**	UNIVPM	ECe308		**10.18730/H84MU**
**SBP123**	UNIVPM	ECe312		**10.18730/H84R3**
**SBP125**	UNIVPM	ECe321		**10.18730/H851C**
**SBP126**	UNIVPM	ECe323		**10.18730/H853E**
**SBP127**	UNIVPM	ECe333		**10.18730/H85DR**
**SBP129**	VILMORIN	79	ARRAS	**10.18730/SC2CT**
**SBP130**	VILMORIN	1636	BAMAKO	**10.18730/SC2DV**
**SBP131**	VILMORIN	181	BOOSTER	**10.18730/SC2EW**
**SBP132**	VILMORIN	2974	BORSALINO	**10.18730/SC2FX**
**SBP133**	VILMORIN	2896	CAPRICE	**10.18730/SC2GY**

**SBP135**	VILMORIN	402	DELINEL	**10.18730/SC2HZ**
**SBP136**	VILMORIN		CROSSER	**10.18730/SC2J***
**SBP137**	VILMORIN		DENVER	**10.18730/SC2K~**
**SBP138**	VILMORIN		DEXTER	**10.18730/SC2M$**
**SBP140**	VILMORIN	2838	ECLAREL	**10.18730/SC2N=**
**SBP141**	VILMORIN	3061	EXPER	**10.18730/SC2PU**
**SBP142**	VILMORIN		FLANKER	**10.18730/SC2Q0**
**SBP143**	VILMORIN	661	GOLDITO	**10.18730/SC2R1**
**SBP144**	VILMORIN	3188	GOURMANDEL	**10.18730/SC2S2**
**SBP145**	VILMORIN	3181	JOGGER	**10.18730/SC2T3**
**SBP146**	VILMORIN	3014	JUMPER	**10.18730/SC2V4**
**SBP147**	VILMORIN	3003	KAISER	**10.18730/SC2W5**
**SBP148**	VILMORIN	3308	KIFER	**10.18730/SC2X6**
**SBP150**	VILMORIN	2046	OREO	**10.18730/SBC9P**
**SBP151**	VILMORIN	1102	OXINEL	**10.18730/SC2Y7**
**SBP152**	VILMORIN	1169	POLDER	**10.18730/SC2Z8**
**SBP153**	VILMORIN	1184	PRIMEL	**10.18730/SC309**
**SBP154**	VILMORIN		PUNCHER	**10.18730/SC31A**
**SBP155**	VILMORIN	1990	REPORTER	**10.18730/SC32B**
**SBP156**	VILMORIN		ROADSTER	**10.18730/SC33C**
**SBP157**	VILMORIN	3024	SORIA	**10.18730/SC34D**
**SBP158**	VILMORIN		SUNDANCE	**10.18730/SC35E**
**SBP160**	VILMORIN	909	TAUSI	**10.18730/SC36F**
**SBP161**	VILMORIN	3158	VANILLA	**10.18730/SC37G**
**SBP162**	VILMORIN	1661	VELERO	**10.18730/SC38H**
**SBP163**	VILMORIN		VEZER	**10.18730/SC39J**
**SBP164**	VILMORIN		WALKER	**10.18730/SBCBR**
**SBP165**	VILMORIN		CALYPSO	**10.18730/SC3AK**
**SBP166**	VILMORIN	868	MARBEL	**10.18730/SC3BM**
**SBP167**	VILMORIN	1366	SKIPPER	**10.18730/SC3CN**
**SBP168**	VILMORIN	1513	VILBEL	**10.18730/SC3DP**
**SBP169**	INRA_PARIS	15	ALLEGRO	**10.18730/SC3EQ**
**SBP173**	INRA_PARIS	28	BRELAN	**10.18730/SC3FR**
**SBP174**	INRA_PARIS	31	CARLIN	**10.18730/SC3GS**
**SBP175**	INRA_PARIS	52	FRONTA	**10.18730/SC3HT**
**SBP177**	INRA_PARIS	64	GREEN ISLE	**10.18730/SC3JV**
**SBP178**	INRA_PARIS	65	GREEN TOP	**10.18730/SC3KW**
**SBP179**	INRA_PARIS	66	GRIS DEUIL	**10.18730/SC3MX**
**SBP180**	INRA_PARIS	77	IMPROVED_SUPER	**10.18730/SC3NY**
**SBP182**	INRA_PARIS	91	LARO	**10.18730/SC3PZ**
**SBP183**	INRA_PARIS	99	LUMINEL	**10.18730/SC3Q***

**SBP184**	INRA_PARIS	100	MARGELIN	**10.18730/SC3R~**
**SBP186**	INRA_PARIS	105	MITARNEL	**10.18730/SC3S$**
**SBP187**	INRA_PARIS	111	NOBLESSA	**10.18730/SC3T=**
**SBP188**	INRA_PARIS	112	OKTAVO	**10.18730/SC3VU**
**SBP189**	INRA_PARIS	116	PASTEL	**10.18730/SC3W0**
**SBP190**	INRA_PARIS	117	PEPITA	**10.18730/SC3X1**
**SBP191**	INRA_PARIS	118	PICO	**10.18730/SC3Y2**
**SBP192**	INRA_PARIS	119	PIROL	**10.18730/SC3Z3**
**SBP193**	INRA_PARIS	120	PISSARO	**10.18730/SC404**
**SBP194**	INRA_PARIS	121	PRELUDE SANS FIL	**10.18730/SC415**
**SBP195**	INRA_PARIS	124	PRESIDENT	**10.18730/SC426**
**SBP197**	INRA_PARIS	136	RONDELLE	**10.18730/SC437**
**SBP198**	INRA_PARIS	137	SABA	**10.18730/SC448**
**SBP200**	INRA_PARIS	139	SAXARES	**10.18730/SC459**
**SBP201**	INRA_PARIS	144	SIMARES	**10.18730/SC46A**
**SBP203**	INRA_PARIS	325	DE MORIONVILLE	**10.18730/SC47B**
**SBP204**	INRA_PARIS	376	GILADEAU	**10.18730/SC48C**
**SBP209**	INRA_PARIS	437	PIERROT	**10.18730/SC49D**
**SBP210**	INRA_PARIS	438	PITAR	**10.18730/SC4AE**
**SBP211**	INRA_PARIS	441	PRINCO	**10.18730/SC4BF**
**SBP212**	INRA_PARIS	442	PRINLADE	**10.18730/SC4CG**
**SBP213**	INRA_PARIS	443	PRINSTALITE	**10.18730/SC4DH**
**SBP217**	INRA_PARIS	466	SLENDERGREEN	**10.18730/SC4EJ**
**SBP219**	INRA_PARIS	479	ALMERE	**10.18730/SC4FK**
**SBP220**	INRA_PARIS	480	ALTERNOR	**10.18730/SC4GM**
**SBP221**	INRA_PARIS	487	BELAMI	**10.18730/SC4HN**
**SBP223**	INRA_PARIS	497	HARVESTER	**10.18730/SBC2F**
**SBP224**	INRA_PARIS	501	IPRIN	**10.18730/SC4JP**
**SBP226**	INRA_PARIS	509	PETRA	**10.18730/SC4KQ**
**SBP227**	INRA_PARIS	510	PHOENIX	**10.18730/SC4MR**
**SBP228**	INRA_PARIS	511	PLUTEX	**10.18730/SC4NS**
**SBP230**	INRA_PARIS	515	PRESTINOE	**10.18730/SC4PT**
**SBP231**	INRA_PARIS	516	SALIA	**10.18730/SC4QV**
**SBP232**	INRA_PARIS	517	SCORE	**10.18730/SC4RW**
**SBP233**	INRA_PARIS	518	SILVERT	**10.18730/SC4SX**
**SBP234**	INRA_PARIS	519	SIMONEX	**10.18730/SC4TY**
**SBP235**	INRA_PARIS	534	MAXIDOR	**10.18730/SC4VZ**
**SBP236**	INRA_PARIS	536	ORNEL	**10.18730/SC4W***
**SBP237**	INRA_PARIS	538	SEMIDOR	**10.18730/SC4X~**
**SBP239**	INRA_PARIS	552	MENIL	**10.18730/SC4Y$**
**SBP240**	INRA_PARIS	559	PRENEL	**10.18730/SC4Z=**

**SBP242**	INRA_PARIS	561	ROMULUS	**10.18730/SC50U**
**SBP243**	INRA_PARIS	568	ARAMIS	**10.18730/SC510**
**SBP244**	INRA_PARIS	569	JANUS	**10.18730/SC521**
**SBP245**	INRA_PARIS	572	TALISMAN	**10.18730/SC532**
**SBP246**	INRA_PARIS	587	FRINGANT	**10.18730/SC543**
**SBP248**	INRA_PARIS	595	MIRY	**10.18730/SC554**
**SBP249**	INRA_PARIS	597	PURENEL	**10.18730/SC565**
**SBP250**	INRA_PARIS	599	RELIMAX	**10.18730/SC576**
**SBP251**	INRA_PARIS	600	SACONEL	**10.18730/SC587**
**SBP252**	INRA_PARIS	602	SAXA	**10.18730/SC598**
**SBP253**	INRA_PARIS	641	MICHENEL	**10.18730/SC5A9**
**SBP255**	**GAUTIER**	PV0953	NEGRITAL	**10.18730/SC5BA**
**SBP256**	**GAUTIER**	PV1340	AIGUILLON	**10.18730/SC5CB**
**SBP257**	**GAUTIER**	PV1341	KRYPTON	**10.18730/SC5DC**
**SBP258**	**GAUTIER**	PV1342	GALION	**10.18730/SC5ED**
**SBP259**	**GAUTIER**	PV1343	PONGO	**10.18730/SC5FE**
**SBP260**	**GAUTIER**	PV1343	VESPERAL	**10.18730/SC5GF**
**SBP262**	**GAUTIER**	PV1345	IRAGO	**10.18730/SC5HG**
**SBP263**	**GAUTIER**	PV1346	TALGO	**10.18730/SC5JH**
**SBP264**	**GAUTIER**	PV1347	STENTOR	**10.18730/SC5KJ**
**SBP265**	**GAUTIER**	PV1348	MAJOR	**10.18730/SC5MK**
**SBP266**	**GAUTIER**	PV1350	PASTORAL	**10.18730/SC5NM**
**SBP267**	**GAUTIER**	PV1351	ROBIN	**10.18730/SC5PN**
**SBP268**	**GAUTIER**	PV1352	ZEPELIN	**10.18730/SC5QP**
**SBP269**	UNIVPM		AR_ARNO	**10.18730/SC5RQ**
**SBP270**	UNIVPM		MINIDOR	**10.18730/SC5SR**
**SBP271**	UNIVPM		GINEVRA	**10.18730/SC5TS**
**SBP273**	UNIVPM		GRENADA	**10.18730/SC5VT**
**SBP274**	UNIVPM		TEMA	**10.18730/SC5WV**
**SBP276**	SERIDA		SUPERNANO GIALLO	**10.18730/SC5XW**
**SBP277**	VRDS Bacau	RO_1		**10.18730/SC5YX**
**SBP278**	VRDS Bacau	RO_2	MIRUNA	**10.18730/SC5ZY**
**SBP279**	VRDS Bacau	RO_3		**10.18730/SC60Z**
**SBP280**	VRDS Bacau	RO_4		**10.18730/SC61***
**SBP283**	VRDS Bacau	RO_7		**10.18730/SC62~**
**SBP284**	VRDS Bacau	RO_8	SALVICA	**10.18730/SC63$**
**SBP285**	VRDS Bacau	RO_9		**10.18730/SC64=**
**SBP287**	SERIDA		WIDUSA	**10.18730/SBC4H**
**SBP288**	SERIDA	S214	LA VICTORIE	**10.18730/SBB9V**
**SBP289**	SERIDA	S097	TENDERGREEM	**10.18730/SBBV8**

**SBP291**	SERIDA	S151	IMPROVED TENDERGREEM	**10.18730/SC65U**
**SBP292**	SERIDA	S149	TOPCROP	**10.18730/SC660**
**SBP293**	UNIVPM		MESSI	**10.18730/SC671**
**SBP295**	UNIVPM		MOONSTONE	**10.18730/SC682**
**SBP296**	UNIVPM		BALTIMORE	**10.18730/SC693**
**SBP297**	UNIVPM		CHICA	**10.18730/SC6A4**
**SBP298**	UNIVPM		KYSIA	**10.18730/SC6B5**
**SBP299**	UNIVPM		ORIENTE	**10.18730/SC6C6**
**SBP300**	UNIVPM		RIMEMBER	**10.18730/SC6D7**
**SBP301**	UNIVPM		SYBARIS	**10.18730/SC6E8**
**SBP302**	UNIVPM	SV1541GA		**10.18730/SC6F9**
**SBP303**	AIS	PHA0001/SRGB00001		**10.18730/SC6GA**
**SBP304**	AIS	PHA0003/SRGB00003		**10.18730/SC6HB**
**SBP305**	AIS	PHA0005/SRGB00005		**10.18730/SC6JC**
**SBP306**	AIS	PHA0011/SRGB00012		**10.18730/SC6KD**
**SBP307**	AIS	PHA0015/SRGB00016		**10.18730/SC6ME**
**SBP308**	AIS	PHA0043/SRGB00044		**10.18730/SC6NF**
**SBP311**	AIS	PHA0120/SRGB00122		**10.18730/SC6PG**
**SBP312**	AIS	PHA0126/SRGB00128		**10.18730/SC6QH**
**SBP314**	AIS	PHA0198/SRGB00200		**10.18730/SC6RJ**
**SBP315**	AIS	PHA0201/SRGB00203		**10.18730/SC6SK**
**SBP316**	AIS	PHA0202/SRGB00204		**10.18730/SC6TM**
**SBP317**	AIS	PHA0213/SRGB00215		**10.18730/SC6VN**
**SBP318**	AIS	PHA0215/SRGB00217		**10.18730/SC6WP**
**SBP319**	AIS	PHA0266/SRGB00267		**10.18730/SC6XQ**
**SBP320**	AIS	PHA0295/SRGB00290		**10.18730/SC6YR**
**SBP321**	AIS	PHA0307/SRGB00302		**10.18730/SC6ZS**
**SBP322**	AIS	PHA0311/SRGB00306		**10.18730/SC70T**
**SBP324**	AIS	PHA0332/SRGB00327		**10.18730/SC71V**
**SBP325**	AIS	PHA0356/SRGB00351		**10.18730/SC72W**
**SBP326**	AIS	PHA0424/SRGB00419		**10.18730/SC73X**
**SBP328**	AIS	PHA0477/SRGB00469		**10.18730/SC74Y**
**SBP329**	AIS	PHA0479/SRGB00471		**10.18730/SC75Z**
**SBP330**	AIS	PHA0498/SRGB00487		**10.18730/SC76***
**SBP331**	AIS	PHA0553/SRGB00524		**10.18730/SC77~**
**SBP332**	AIS	PHA0670/SRGB00632		**10.18730/SC78$**
**SBP333**	AIS	PHA0671/SRGB00633		**10.18730/SC79=**
**SBP334**	AIS	PHA0674/SRGB00635		**10.18730/SC7AU**
**SBP335**	AIS	PHA0687/SRGB00644		**10.18730/SC7B0**
**SBP336**	AIS	PHA0690/SRGB00647		**10.18730/SC7C1**

**SBP340**	AIS	PHA0931/SRGB00867		**10.18730/SC7D2**
**SBP341**	AIS	PHA0956/SRGB00892		**10.18730/SC7E3**
**SBP343**	AIS	PHA0997/SRGB00932		**10.18730/SC7F4**
**SBP344**	AIS	PHA1009/SRGB00944		**10.18730/SC7G5**
**SBP345**	AIS	PHA1015/SRGB00950		**10.18730/SC7H6**
**SBP346**	AIS	PHA1020/SRGB00955		**10.18730/SC7J7**
**SBP347**	AIS	PHA1036/SRGB00971		**10.18730/SC7K8**
**SBP350**	PSR	GE-676	MARCHÉ DE VULLY	**10.18730/SC7M9**
**SBP351**	PSR	GE-1023	ROI DES BEURRES	**10.18730/SC7NA**
**SBP352**	PSR	GE-1174	FIN DE BAGNOLS	**10.18730/SC7PB**
**SBP355**	PSR	GE-1336	SLENDERWAX	**10.18730/SC7QC**
**SBP356**	PSR	PSR-100007	WUNDERFEIN SELEKTION Z	**10.18730/SC7RD**
**SBP357**	PSR	GE-1312	ODESSA (WUNDERFEIN)	**10.18730/SC7SE**
**SBP358**	PSR	GE-1162	BENEVENTO	**10.18730/SC7TF**
**SBP359**	PSR	GE-717	BROWN SWISS	**10.18730/SC7VG**
**SBP360**	PSR	GE-569	EINKORNBOHNE	**10.18730/SC7WH**
**SBP361**	PSR	GE-587	ENFANT DE MONT CALME	**10.18730/SC7XJ**
**SBP362**	PSR	GE-973	EMPEREUR DE RUSSIE	**10.18730/SC7YK**
**SBP363**	PSR	GE-1042	GARTENGLÜCK	**10.18730/SC7ZM**
**SBP364**	PSR	GE-971	GOLDEN BUTTER	**10.18730/SC80N**
**SBP365**	PSR	GE-681	GELBHÜLSIGE	**10.18730/SC81P**
**SBP366**	PSR	GE-3169	HUNDERT FÜR EINE	**10.18730/SC82Q**
**SBP367**	PSR	GE-1384	IDEAL DIETIKON	**10.18730/SC83R**
**SBP369**	PSR	GE-1034	MARCHÉ DES TROIS CHÊNES	**10.18730/SC84S**
**SBP371**	PSR	GE-588	MARCHÉ DE GENÈVE	**10.18730/SC85T**
**SBP372**	PSR	GE-28	MAKEDONISCHE	**10.18730/SC86V**
**SBP373**	PSR	GE-953	OKTOBERLI	**10.18730/SC87W**
**SBP374**	PSR	GE-972	RED VALENTINE	**10.18730/SC88X**
**SBP375**	PSR	GE-1016	PROCESSOR ORDER SABO	**10.18730/SC89Y**
**SBP377**	PSR	GE-789	VON BOSWIL	**10.18730/SC8AZ**
**SBP378**	PSR	GE-643	WICHINGEN	**10.18730/SC8B***
**SBP381**	PSR	GE-3202	RABE	**10.18730/SC8C~**
**SBP382**	PSR	GE-3565	PETIT RIZ	**10.18730/SC8D$**

Donor (1)

**AIS**	AGRICULTURAL INSTITUTE OF SLOVENIA
**GAUTIER**	GAUTIER_SEMENCES
**INRA**	INSTITUT NATIONAL DE LA RECHERCHE AGRONOMIQUE
**PSR**	PROSPECIERARA

**SERIDA**	AGRI-FOOD RESEARCH AND DEVELOPMENT REGIONAL SERVICE, ASTURIAS, SPAIN
**UNIVPM**	UNIVERSITÀ POLITECNICA DELLE MARCHE, ANCONA, ITALY
**VRDS Bacau**	STATIUNEA DE CERCETARE DEZVOLTARE PENTRU LEGUMICULTURA BACAU

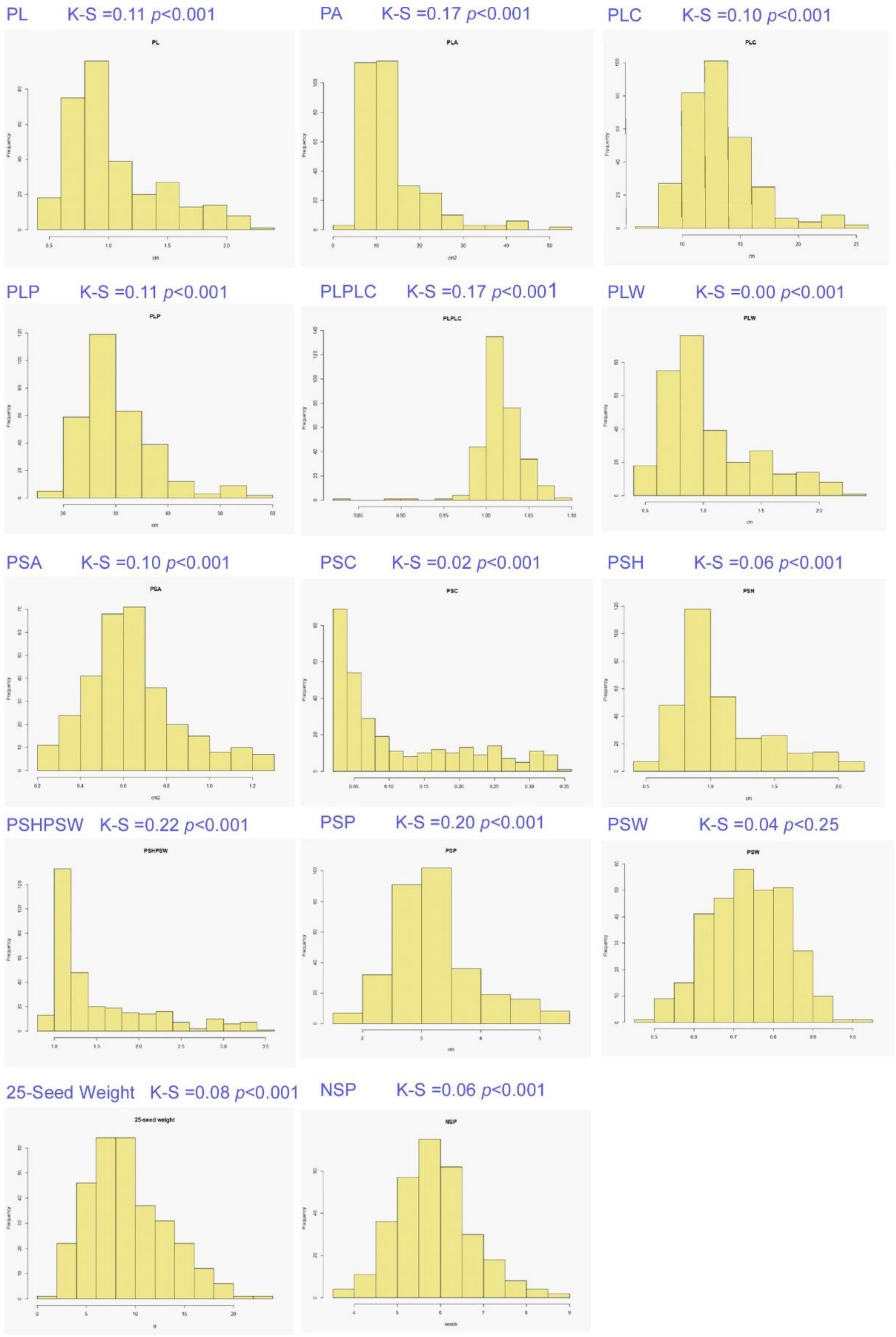

Figure S1. Histograms showing the distributions (mean of two environments data adjusted with LSmeans) for the 14 quantitative pod traits assessed in the Snap Diversity Panel. Kolmogorov-Smirnov normality test (K-S)

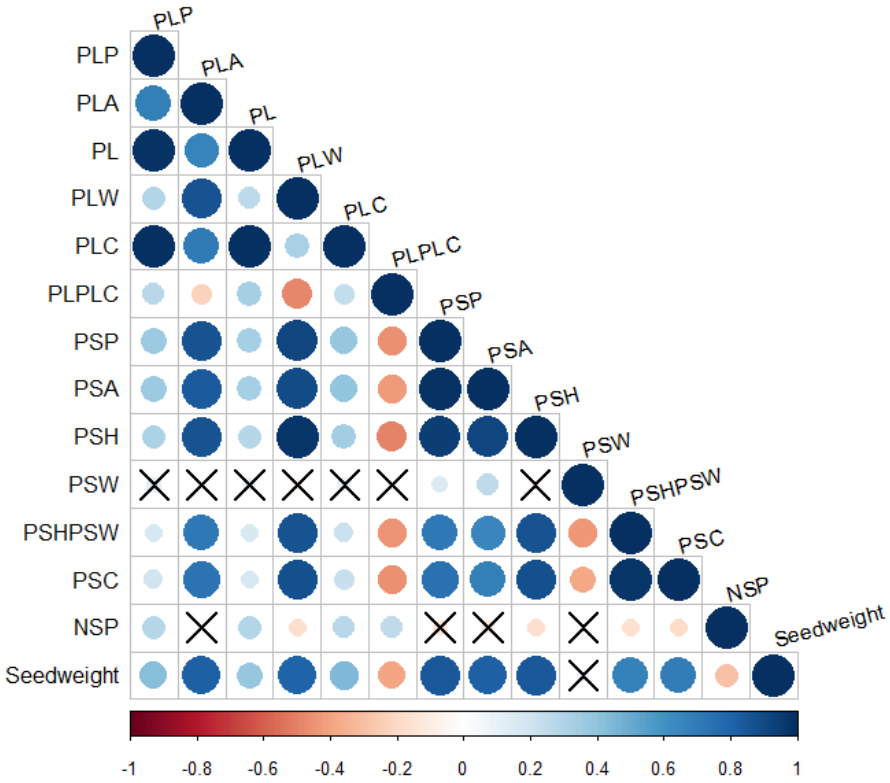

Figure S2. Corrplot showing the Spearman correlation among the 14 quantitative pod traits evaluated. Non-significant correlations ($\alpha = 0.05$) are indicated with 'X'.

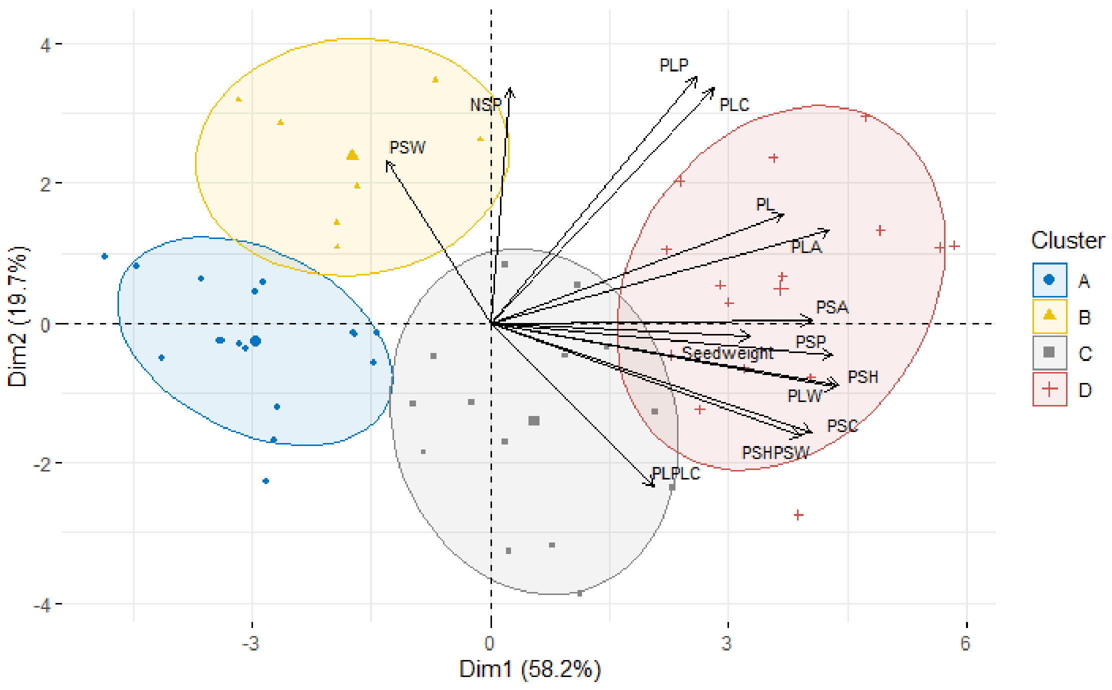

Figure S3. Biplots showing the Hierarchical Clustering results on Principal Components analysis from 14 evaluated quantitative pod traits in the Core-SBP. Ellipses representing the clusters were drawn considering a confidence interval > 0.8.

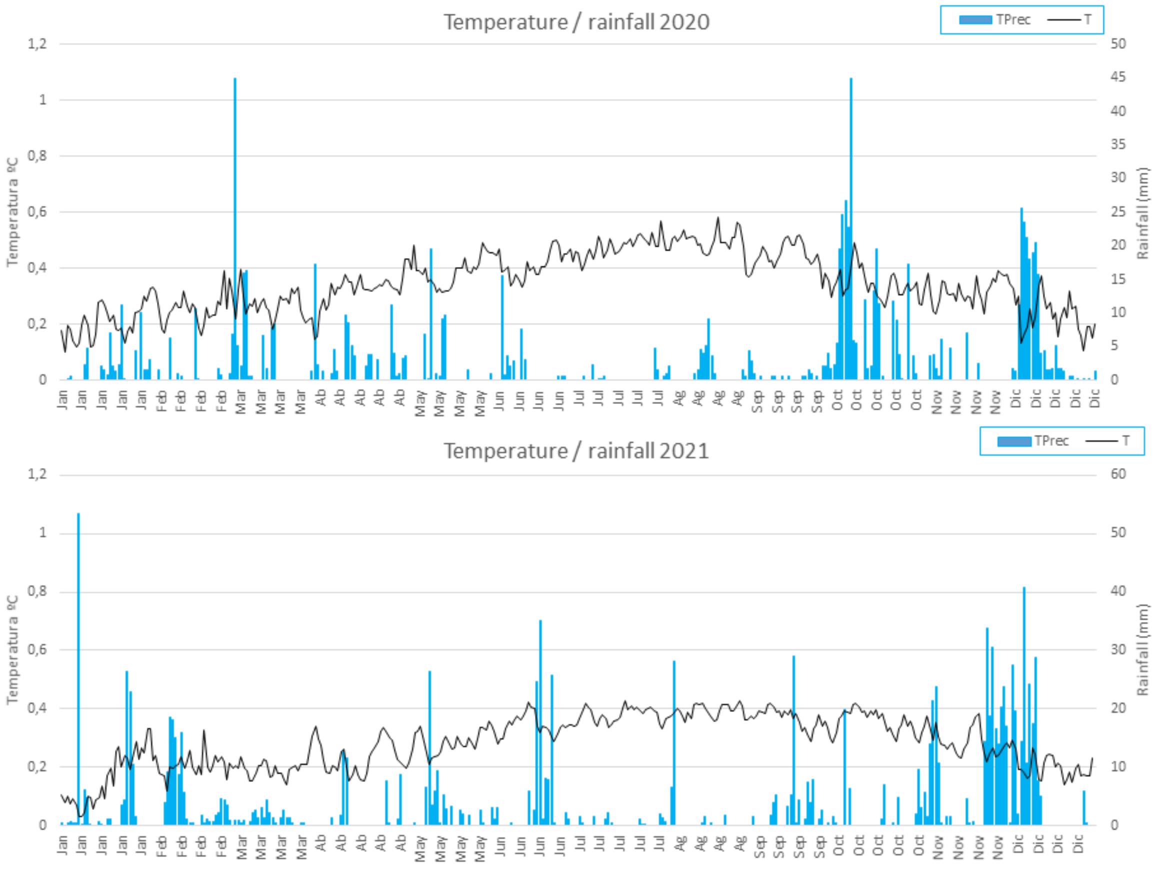

Figure S4. Temperature (daily average in ºC) and rainfall (mm) were recorded in the field crops during 2020 and 2021. The crops were carried out between May and September.
